# Development and Validation of a Culturally Competent Care Module and Its Effect on Nurses’ Cultural Competence and Patient Satisfaction: Protocol for a Randomized Controlled Trial in a Tertiary Care Hospital in India

**DOI:** 10.2196/84015

**Published:** 2026-07-29

**Authors:** Ligy C Ittup, Ranjana Sharma

**Affiliations:** 1 Smt. Radhikabai Meghe Memorial College of Nursing Datta Meghe Institute of Higher Education and Research (Deemed to be University) Wardha, Maharashtra India; 2 Holy Spirit Hospital Mumbai, Maharashtra India; 3 Shalinitai Meghe College of Nursing Datta Meghe Institute of Higher Education and Research (Deemed to be University) Wardha, Maharashtra India

**Keywords:** cultural competence, nursing care, patient satisfaction, randomized controlled trial, nursing education

## Abstract

**Background:**

Culturally responsive nursing involves delivering care that respects and reflects patients’ cultural values, communication patterns, and belief systems. In the Indian context, increasing rural-to-urban migration along with the expansion of medical tourism, estimated to have attracted 2.1 million international patients in 2024, has intensified the need for culturally competent nursing care. Although educational interventions in this area have shown encouraging results, there remains a lack of well-designed randomized controlled trials (RCTs) in India. For the purpose of this study, structured training is understood as a planned, session-based approach to learning that includes activities such as simulation exercises, case-based discussions, and guided reflection.

**Objective:**

This study aims to develop, validate, and evaluate a culturally competent care module (CCCM) through a 3-phase RCT, assessing its effects on nurses’ cultural competence and patient satisfaction.

**Methods:**

This 3-phase parallel-group RCT is being conducted in the medical-surgical wards of the Acharya Vinoba Bhave Rural Hospital, Wardha, Maharashtra, India (2024-2026). In phase 1 (completed), the CCCM was developed via thematic analysis of 20 interviews with nurses, educators, and patients. In phase 2 (completed), the CCCM and a patient satisfaction tool were validated using a 2-round Delphi process involving 15 experts (CCCM: content validity index ≥0.89; patient satisfaction tool: Cronbach α=0.87-0.92). Phase 3 is ongoing: 55 nurses are randomized in a 1:1 ratio to either the CCCM (5×1-hour sessions+boosters) or routine care. The primary outcome is the postintervention Cultural Competence Assessment Tool for Nurses score, while secondary outcomes include subscales, patient satisfaction, and 90-day retention. The sample size is calculated using G*Power based on an anticipated effect size of 0.82. Participants are randomly allocated through computer-generated block randomization stratified by seniority, and outcome assessors and data analysts are blinded to group allocation. Data will be analyzed using SPSS (intention-to-treat, linear mixed-effects models; *P*<.05). Patient satisfaction outcomes will be evaluated using data from 120 patients.

**Results:**

Phases 1 and 2 were completed in July 2025. In phase 3, 110 (100%) nurses were successfully enrolled and randomized, with 55 participants assigned to each group. The intervention was delivered as planned, and postintervention data collection reached 70% completion. Additionally, of the planned 120 patients, 92 (77%) were enrolled for the patient satisfaction assessment. Full data analysis is expected to be completed by June 2026.

**Conclusions:**

This trial will provide evidence on the CCCM’s noninferiority or superiority to routine care for enhancing cultural competence and satisfaction, supporting scalable training amid India’s demographic shifts.

**Trial Registration:**

Clinical Trials Registry–India (CTRI) CTRI/2025/06/088982; https://ctri.nic.in/Clinicaltrials/pmaindet2.php?EncHid=MTMzMjQ5&Enc=&userName=

**International Registered Report Identifier (IRRID):**

DERR1-10.2196/84015

## Introduction

Culturally responsive nursing practice extends beyond routine clinical competence and requires the ability to deliver care that is consistent with patients’ cultural values, communication patterns, spiritual beliefs, and health practices [1]. In nursing literature, cultural competence is not viewed as a fixed end point but as a continuous and evolving process. Campinha-Bacote’s [2] process model conceptualizes this progression through 5 interrelated constructs: cultural awareness, cultural knowledge, cultural skill, cultural encounters, and cultural desire. The model highlights the importance of reflective practice, experiential learning, and ongoing engagement with culturally diverse populations.

The growing need for cultural competence in India is closely linked to ongoing demographic transitions. Rapid rural-to-urban migration has diversified patient populations in metropolitan and semiurban health care settings [3]. At the same time, India has become a prominent destination for medical value travel, with approximately 2.1 million international patients reported in 2024 [4]. This trend reflects broader global patterns, with more than 300 million international migrants documented worldwide by mid-2024 [5]. These changes have made culturally responsive care an essential component of quality health care delivery.

Evidence from international settings demonstrates the effectiveness of structured cultural competence training. In this context, structured training refers to formally designed, curriculum-based educational interventions that include clearly defined learning objectives; standardized content; and guided learning strategies such as simulation exercises, case-based discussions, role-play, and reflective practice sessions. For example, simulation-based intercultural scenarios used in the United States and case-based training modules integrated into continuing education programs in Europe have demonstrated improvements in communication skills and culturally appropriate clinical decision-making. A recent randomized trial (2025) further reported significant improvements in cultural knowledge and sensitivity following structured educational intervention [2]. A multicenter Ethiopian study also identified a positive association between nurses’ cultural competence and equitable health care delivery [6], while a contemporary scoping review concluded that structured educational modules strengthen communication and responsiveness to patients’ needs [7].

Within the Indian context, available evidence suggests progress but also notable limitations. Quasi-experimental studies have reported improvements in nurse-patient communication following targeted training interventions [8]. However, challenges such as linguistic diversity and religious plurality continue to influence care delivery [9]. Findings from medical tourism settings indicate that perceived cultural insensitivity can negatively affect patients’ trust and satisfaction [10]. Despite these insights, a key limitation in Indian research is the lack of rigorously designed, context-specific interventions evaluated through robust methodologies. Most studies rely on quasi-experimental designs and do not assess long-term retention of skills. Research from rural Maharashtra further highlights a disconnect between awareness and actual practice of cultural competence [11].

In response to these gaps, this study proposes the development and evaluation of a culturally competent care module (CCCM) tailored for practicing nurses. The module is designed as a structured educational intervention aimed at improving both cultural competence and patient-centered care outcomes.

The conceptual foundation of the CCCM draws on established theoretical frameworks, including Campinha-Bacote’s [2] model, Giger and Davidhizar’s [12] transcultural assessment model, and Purnell’s [13] model for cultural competence. In addition, organizational perspectives emphasize the role of cultural alignment in enhancing health care delivery and patient outcomes [14]. International policy guidance, such as the World Health Organization’s framework on intercultural health care, further supports the integration of cultural competence training to address health inequities [15].

Systematic reviews indicate that structured cultural competence training improves communication outcomes and patient satisfaction when compared with routine care [16]. However, rigorously designed randomized controlled trials (RCTs) in India that assess both nurse-level and patient-level outcomes remain limited.

This study therefore aims to develop, validate, and evaluate the CCCM using a RCT design. It is hypothesized that nurses who receive CCCM training will demonstrate improved cultural competence (Cultural Competence Assessment Tool for Nurses [CCAT-N] scores; Multimedia Appendix 1) and that their patients will report greater satisfaction compared to those receiving standard care.

## Methods

### Study Design

This study is a 3-phase, parallel-group RCT with individual-level allocation. It is being conducted between 2025 and 2026 in the medical-surgical wards of the Acharya Vinoba Bhave Rural Hospital, Wardha, Maharashtra, India. Phases 1 and 2 focused on the development and validation of the CCCM and have been completed. This protocol describes phase 3, which evaluates the effectiveness of the CCCM through a randomized comparison.

### Study Setting

The trial is being conducted in 6 independent medical-surgical wards of the Acharya Vinoba Bhave Rural Hospital, a tertiary care teaching hospital serving a culturally and socioeconomically diverse population from rural and semiurban regions of Maharashtra. Each ward is staffed by 2 to 3 registered nurses per shift, which supports individual randomization while reducing the risk of contamination between study groups.

### Participants

A total of 110 registered nurses are being recruited. Eligible participants are nurses currently working in medical-surgical wards with at least 1 year of clinical experience. Written informed consent is obtained prior to participation. Nurses who have previously completed formal training in cultural competence are excluded to minimize bias related to prior exposure.

### Sample Size Determination

The sample size is calculated based on the primary outcome of cultural competence measured using the CCAT-N. Pilot data indicated a large effect size (Cohen *d*≈0.8). Assuming a 2-tailed significance level of .05 and a statistical power of 90%, a minimum of 34 participants per group is required. After accounting for a potential attrition rate of 20%, the final sample size is set at 55 participants per group, resulting in a total of 110 nurses.

### Randomization and Blinding

Participants are randomly assigned in a 1:1 ratio to the intervention or control group using a block randomization approach with stratification by ward seniority (junior vs senior nurses). Allocation concealment is ensured using sequentially numbered, opaque, sealed envelopes prepared independently before enrollment. Blinding of participants is not feasible due to the nature of the educational intervention. However, outcome assessors and data analysts remain blinded to group allocation. To reduce contamination, training sessions are conducted during off-duty hours, intervention materials are restricted to the intervention group, and patient allocation is organized after completion of training. The control group is offered access to the CCCM after study completion.

### Study Phases

#### Phase 1

The CCCM was developed using a qualitative exploratory design. In-depth interviews were conducted with registered nurses, nurse educators, sociologists, psychologists, and patients from diverse cultural backgrounds (Multimedia Appendix 2). The interviews explored communication barriers, culturally sensitive practices, and sociocultural considerations in care delivery. Data were analyzed using Braun and Clarke’s [17] 6-step thematic analysis framework. Emergent themes informed the structure, learning objectives, and content domains of the CCCM.

#### Phase 2

Validation of the CCCM and patient satisfaction tool: the CCCM and a culturally sensitive patient satisfaction tool were validated using a 2-round Delphi process involving experts in transcultural nursing and health care communication. Content validity was assessed in terms of relevance, clarity, and applicability. The CCCM achieved a content validity index (CVI) of ≥0.89. Revisions were incorporated based on expert feedback.

Phases 1 and 2 of the study are completed. Phase 3 is planned as per the following details.

#### Phase 3

RCT: participants are randomized to either the intervention group (CCCM plus routine care) or the control group (routine care only). The effectiveness of the intervention is assessed through comparison of outcomes between groups.

### Intervention

The CCCM is a structured educational intervention designed to improve cultural competence among nurses. It is delivered as five 1-hour sessions over 5 consecutive days, followed by booster sessions on days 15, 35, and 45. The module includes components on cultural self-awareness (guided by the ASKED [awareness, skills, knowledge, encounter, desire] framework), cross-language communication, religious and end-of-life care, and sociocultural considerations in the Indian context. It also incorporates elements of leadership and advocacy in culturally responsive practice. The components of the CCCM were systematically mapped to established cultural competence frameworks to ensure theoretical grounding and comprehensiveness, as shown in Table 1.

A blended learning approach is used, combining didactic teaching with experiential methods such as simulation, role-play, and case-based discussions. Participants are required to attend at least 80% of sessions to be considered compliant. The control group continues routine nursing care without exposure to the CCCM during the study period.

**Table 1:** Mapping of culturally competent care module (CCCM) components to cultural competence frameworks.

CCCM session component and Campinha-Bacote [2] domains			Purnell [13] domains		Giger and Davidhizar [12] phenomena
Cultural self-awareness (ASKED^a^ framework)					
	Cultural awareness	Overview or heritage		Social organization	
	Cultural desire	Workforce issues		Environmental control	
Cross-language communication strategies					
	Cultural skill	Communication		Communication	
	Cultural encounters	Health care practices		Space	
Religious and end-of-life considerations					
	Cultural knowledge	Spirituality		Social organization	
	Cultural skill	Death rituals		Time orientation	
India-specific sociocultural adaptations					
	Cultural knowledge	Family roles		Social organization	
	Cultural encounters	Biocultural ecology		Biological variations	
Leadership and advocacy in culturally responsive care					
	Cultural desire	Health care organization		Environmental control	
	Cultural encounters	Workforce issues		Social organization	
Simulation-based role-play					
	Cultural skill	Multiple integrated domains		Communication	
	Cultural encounters	Multiple integrated domains		Time	
	Cultural encounters	Multiple integrated domains		Social organization	

^a^ASKED: awareness, skills, knowledge, encounter, desire.

### Outcomes

The primary outcome is cultural competence, measured using the CCAT-N, a validated instrument that provides a total score reflecting overall cultural competence. Secondary outcomes include domain-specific subscale scores of cultural competence, patient satisfaction, and retention of cultural competence at 90 days following the intervention. Higher scores on these measures indicate improved outcomes.

### Data Collection

Data are collected at 3 time points: baseline (before the randomization), immediately after completion of the intervention, and at 90 days after the intervention to assess retention. The schedule of enrollment, intervention, and outcome assessments is presented in Table 2.

Cultural competence is assessed using the CCAT-N, while patient satisfaction is measured using a culturally competent nursing care satisfaction survey. Standardized data collection procedures are followed across all time points to ensure consistency and reduce measurement bias.

**Table 2:** Schedule of enrollment, interventions, and assessments (SPIRIT [Standard Protocol Items: Recommendations for Interventional Trials] format).

Study component	Baseline (t-1)	Allocation (0)	Weeks 1-2	Week 3	Day 90
Enrollment procedures					
Eligibility screening	✓				
Informed consent	✓				
Baseline demographic data	✓				
Baseline cultural competence (CCAT-N^a^)	✓				
Randomization		✓			
Intervention group (CCCM^b^)					
5 structured CCCM sessions			✓		
Booster reinforcement activities			✓	✓	
Control group					
Routine ward training			✓		
Outcome assessments					
Cultural competence (CCAT-N)	✓			✓	✓
Patient satisfaction survey				✓	✓
Process evaluation checklist			✓	✓	
Adverse event monitoring			✓	✓	✓

^a^CCAT-N: Cultural Competence Assessment Tool for Nurses.

^b^CCCM: culturally competent care module.

### Data Analysis

Data analysis is conducted to compare outcomes between the intervention and control groups. Descriptive statistics are used to summarize baseline characteristics and outcome variables. Baseline comparability between groups is assessed prior to outcome analysis. For the primary outcome, postintervention CCAT-N scores are compared between groups using a 2-tailed independent samples *t* test. Where baseline differences are present, analysis of covariance is applied to adjust for baseline values. Changes in outcomes over time are analyzed using repeated measures analysis of variance or mixed-effects modeling to account for within-participants variability. Both intention-to-treat and per-protocol analyses are performed to ensure robustness of findings. Missing data are addressed using multiple imputation methods. For patient satisfaction outcomes, either parametric or nonparametric statistical tests are applied depending on data distribution. Subgroup analyses are conducted to explore potential effect modification based on ward seniority. A 2-tailed *P* value of <.05 is considered statistically significant.

### Data Management

All data are stored in secure, encrypted institutional databases. Double-entry verification is used to ensure accuracy. Data quality is monitored regularly, and an audit trail is maintained throughout the study.

### Ethical Considerations

Ethics approval was obtained from the Datta Meghe Institute of Higher Education & Research Institutional Ethics Committee (DMIHER-DU/IEC/2024/52; December 20, 2024). The trial is registered with the Clinical Trial Registry of India (CTRI/2025/06/088982; May 17, 2025). The study involves minimal risk, as it is an educational intervention. Written informed consent will be obtained from all participants prior to enrollment. Participants will be informed of their right to withdraw at any time without penalty. No formal data monitoring committee has been constituted due to the low-risk nature of the study. The study adheres to the Declaration of Helsinki principles, protecting participants’ rights, privacy, and confidentiality.

## Results

### Trial Status and Progress (Updated February 13, 2026)

#### Phase 1: Development of the CCCM

Phase 1 of the study, which was conducted between March and April 2025, focused on the development of the CCCM. The module was developed using a qualitative approach guided by reflexive thematic analysis, as described by Braun and Clarke [17]. This approach emphasized researcher reflexivity, with the principal investigator maintaining a reflexive journal to document positionality and potential cultural biases throughout the analytical process.

A total of 20 in-depth interviews, each lasting 45 to 60 minutes, were conducted with registered nurses, nurse educators, and patients from diverse cultural backgrounds. Data were analyzed through iterative coding and theme development, resulting in the identification of 5 key domains that informed the structure and content of the CCCM. To ensure methodological rigor, multiple strategies were used. Credibility was enhanced through member checking, while dependability was ensured maintaining a comprehensive audit trail. Confirmability was supported through peer debriefing, and transferability was addressed through detailed contextual descriptions of the study setting and participants.

#### Phase 2: Validation of the CCCM and Study Instruments

Phase 2, conducted between June and July 2025, involved the validation of the CCCM and the culturally adapted patient satisfaction tool. Content validation was performed using a 2-round modified Delphi technique involving 15 experts, including sociologists (n=2), psychologist (n=1) physician (n=1), nurse educators (n=8), and clinical nursing leaders (n=3).

The CCCM achieved a CVI of 0.89, while the patient satisfaction tool demonstrated a CVI of 0.92, indicating high agreement among experts regarding relevance and clarity. The CCAT-N, adapted for this study, demonstrated strong psychometric properties. Internal consistency was high (Cronbach α=0.87), and test-retest reliability over a 14-day interval was satisfactory (*r*=0.82). The tool also showed good sensitivity to change, with a pilot study indicating a large effect size (Cohen *d*=0.82). The 15-item patient satisfaction tool, measured on a 5-point Likert scale, demonstrated high internal consistency (Cronbach α=0.89). Construct validity was established through exploratory factor analysis, which identified 2 primary factors accounting for 73% of the total variance, namely, “respect for cultural practices” (42%) and “effective communication” (31%).

#### Phase 3: RCT Implementation Status

Phase 3 of the study is currently in progress. Participant recruitment commenced in August 2025. As of February 2026, a total of 110 participants have been enrolled and randomized, representing 100% of the target sample size. Participants have been equally allocated to the intervention group (n=55) and the control group (n=55).

Baseline data collection was completed between August and October 2025. The CCCM intervention was delivered to the intervention group between October and November 2025, following which immediate postintervention assessments were conducted in November 2025.

Follow-up assessments at 90 days after the intervention are currently underway and are expected to be completed between February and March 2026. Final data analysis is anticipated to be completed by June 2026.

### Mixed Methods Integration

The study adopts a sequential explanatory mixed methods design, wherein qualitative findings from phase 1 inform the quantitative evaluation in phase 3. Integration of findings is achieved through the use of joint interpretive displays, which link CCCM content domains with corresponding subscales of the CCAT-N. This approach enables the generation of comprehensive metainferences by combining statistical outcomes with experiential insights related to intervention implementation.

### Planned Data Analysis

Quantitative analysis for phase 3 will focus on evaluating the effectiveness of the CCCM intervention. The primary outcome, namely, cultural competence as measured by total CCAT-N scores, will be analyzed using linear mixed-effects models, incorporating random intercepts to account for clustering at the ward level.

Secondary outcomes will be analyzed using generalized estimating equations to assess changes over time across baseline, postintervention, and 90-day follow-up measurements. Mediation analysis will also be conducted to examine whether improvements in cultural competence mediate changes in patient satisfaction.

Missing data will be handled using multiple imputation methods, provided that missingness does not exceed 20%, and patterns of missing data will be carefully examined prior to analysis.

### Qualitative Follow-Up Component

A qualitative component is planned as part of the mixed methods design to further explore participant experiences. Follow-up interviews will be conducted with a purposive sample of 15 nurses and 15 patients. Data will be analyzed using reflexive thematic analysis, with explicit attention to researcher positionality and iterative development of the coding framework.

Trustworthiness of qualitative findings will be ensured using the criteria proposed by Lincoln and Guba [18], including credibility, transferability, dependability, and confirmability. Participant validation will also be undertaken to enhance the authenticity of findings.

### Projected Timeline and Dissemination

The final statistical analysis is expected to be completed between March and June 2026. The findings of the study will be disseminated through peer-reviewed journals focusing on transcultural nursing, nursing education, and health care quality. In addition, the results will be presented at national and international conferences to facilitate wider academic and clinical engagement. Efforts will also be made to share key findings with health care institutions and nursing educators to support the integration of culturally competent practices into clinical training and practice.

## Discussion

### Anticipated Findings

This 3-phase trial hypothesizes that the CCCM will produce moderate-to-large improvements in nurses’ cultural competence and patient satisfaction, consistent with structured intervention studies demonstrating moderate-to-large effect sizes [19,20].

Indian nursing curricula continue to prioritize biomedical competencies over structured transcultural development [21]. These gaps are compounded by limitations in faculty preparedness [22]. Linguistic diversity across India’s 22 officially recognized languages presents persistent communication barriers [23]. These challenges are further amplified by the growth of medical tourism [24]. The CCCM addresses these gaps through a structured 5-domain model grounded in transcultural and cultural safety frameworks [13,25]. Its blended instructional design aligns with simulation-based educational evidence demonstrating superior competency gains [19].

**Figure 1:**
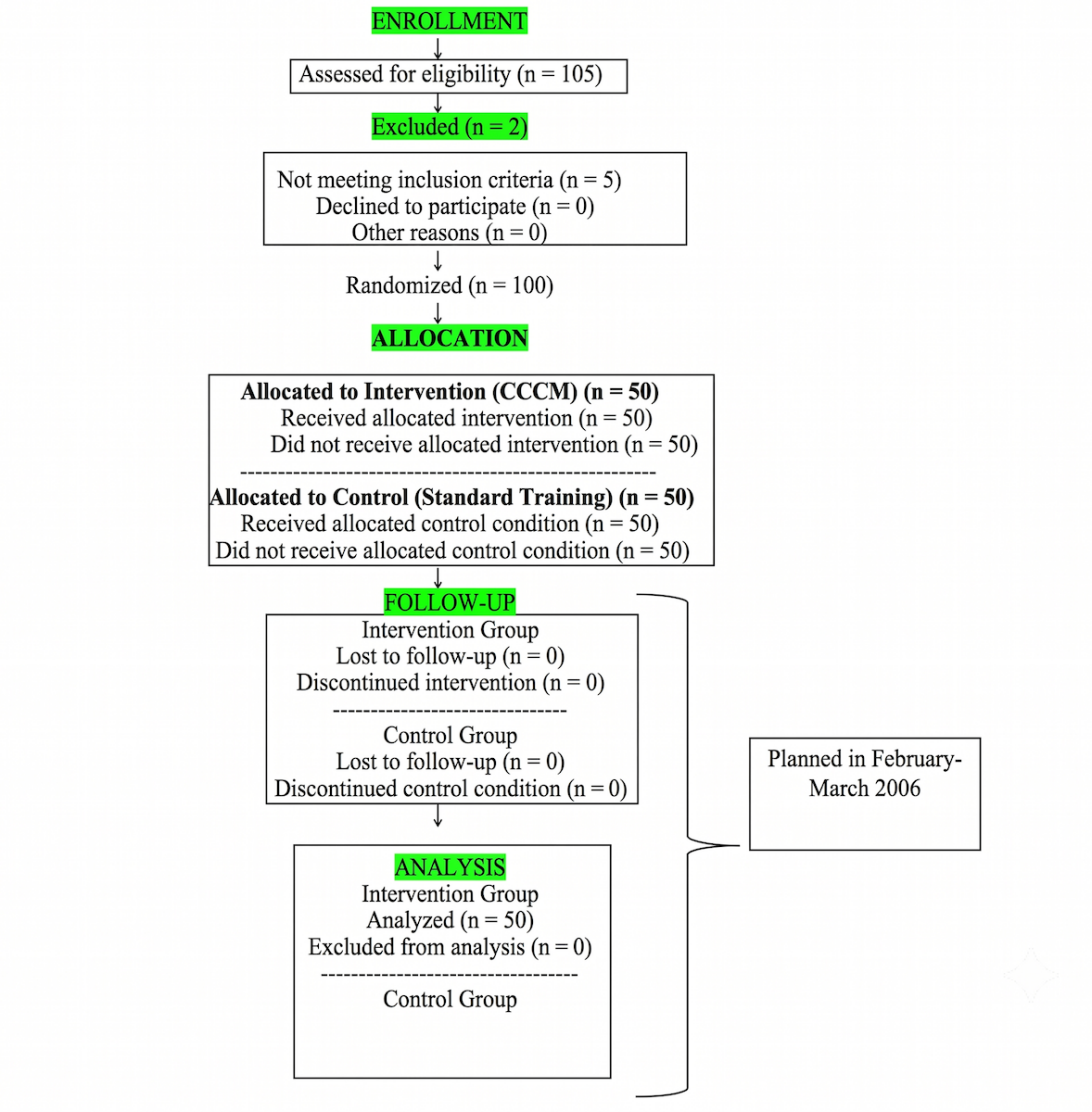
CONSORT (Consolidated Standards of Reporting Trials) 2023 flow diagram—culturally competent care module (CCCM) randomized controlled trial.

Compared with previous evidence from quasi-experimental studies conducted in Indian hospitals [26] and recent RCTs [19], this protocol incorporates CONSORT (Consolidated Standards of Reporting Trials)–aligned randomization (Figure 1) [27], validated psychometric development standards [28], rigorous nursing research methodology [29], structured contamination control strategies, and a 90-day follow-up period. Implementation strategies are informed by updated reach, effectiveness, adoption, implementation, and maintenance (RE-AIM) framework guidance [30]. The single-center setting reflects current migration and linguistic diversity patterns [22]. The selected outcomes are aligned with the World Health Organization’s global health equity priorities [31].

If successful, this trial will provide (1) one of the first rigorously designed RCTs in India evaluating structured cultural competence training; (2) a validated, region-specific assessment and training toolkit; (3) a scalable 5-session intervention model; and (4) an equity-oriented strategy aligned with the World Health Organization’s health equity goals [30].

### Limitations

This study has certain limitations that should be considered when interpreting the findings. First, the trial is conducted in a single tertiary care teaching hospital, which may modestly limit generalizability to other health care settings across India that differ in infrastructure, staffing patterns, patient load, and linguistic diversity.

Second, although contamination control measures were incorporated, some degree of informal knowledge sharing between intervention and control participants in shared clinical environments cannot be entirely ruled out, which may have influenced group differences. Third, selected nurse-level outcomes are based on self-reported measures and may be subject to social desirability tendencies, despite the use of blinded outcome assessment procedures. Fourth, the 3-month follow-up period provides insight into short-term outcomes; however, longer follow-up would offer a clearer understanding of sustainability over time.

Fifth, contextual realities within Indian health care settings—including workload intensity, shift rotations, and limited protected training time—may influence implementation dynamics. These factors should be considered when adapting the intervention to other institutions. Linguistic diversity across regions may also necessitate contextual tailoring of training materials. Finally, while patient satisfaction is included as an outcome indicator, broader structural determinants of health equity extend beyond the scope of this intervention.

### Future Directions

Future research should evaluate the effectiveness of CCCM in multicenter studies involving diverse health care settings across India. Longer follow-up periods are needed to determine the sustainability of improvements in cultural competence and patient satisfaction. Further studies should also examine the implementation of the module in different clinical specialties and evaluate its adaptability to other cultural and health care contexts. In addition, implementation research could explore barriers and facilitators to integrating culturally competent training into routine nursing education and continuing professional development.

### Conclusions

This 3-phase RCT protocol outlines a rigorously designed evaluation of a structured CCCM tailored to the Indian tertiary hospital context. By integrating qualitative module development, expert validation, and a parallel-group randomized design with blinded outcome assessment and follow-up evaluation, the study addresses key methodological gaps identified in recent cultural competence intervention research.

If demonstrated to be effective, the CCCM may provide evidence for a pragmatic, time-efficient, and contextually adapted cultural competence training model capable of improving nurse-level competencies and patient satisfaction outcomes. Given India’s increasing sociolinguistic diversity and expanding international patient population, structured cultural competence training represents both an equity-oriented strategy and a quality improvement initiative. The findings may inform curriculum reform, institutional training policies, and future multicenter effectiveness-implementation hybrid studies.

## Data Availability

Data will be generated and analyzed during phase 3 of this randomized controlled trial. Deidentified participant-level data, including cultural competence scores and patient satisfaction outcomes, will be made available upon reasonable request from the corresponding author following completion of the study and publication of the primary results. Data sharing will be subject to approval by the Institutional Ethics Committee and in accordance with institutional data protection policies to ensure participant confidentiality. Where feasible, aggregated datasets and supporting materials may be made available through publicly accessible repositories or as supplementary files accompanying the publication. Requests for access to the data should include a clear research proposal and will be considered on a case-by-case basis.

## Appendix

Multimedia Appendix 1 Cultural Competence Assessment Tool for Nurses.

Multimedia Appendix 2 Interview format.

## Abbreviations

ASKED: awareness, skills, knowledge, encounter, desire
CCAT-N: Cultural Competence Assessment Tool for Nurses
CCCM: culturally competent care module
CONSORT: Consolidated Standards of Reporting Trials
CVI: content validity index
RCT: randomized controlled trial
