# Supplementary material for: Development and Validation of a Culturally Competent Care Module and Its Effect on Nurses’ Cultural Competence and Patient Satisfaction: Protocol for a Randomized Controlled Trial in a Tertiary Care Hospital in India
Source: JMIR Res Protoc. 2026 Jul 29;15:e84015. doi: 10.2196/84015 (PMC13469827; doi:10.2196/84015)
Supplement: Supplementary file 1 [file resprot_v15i1e84015_app1.docx]

**Cultural Competence Assessment Tool- Nurses (CCAT-N)**

**Demographic Data**

Code No:	Age:	Gender: F/M

Qualification:-GNM/BSc / MSc / PhD

Number of Years in Service:	Department/Unit:

Previous Training on Cultural Competence:

Area of Residence: Rural/ Urban/Semi Urban

**Instructions:**

- **Please indicate your level of agreement with the following statements.**

**Scale:**

**1 – Strongly Disagree | 2 – Disagree | 3 – Neutral | 4 – Agree | 5 – Strongly Agree**

| **Items** | **1** | **2** | **3** | **4** | **5** |
| --- | --- | --- | --- | --- | --- |
|  | **Strongly Disagree** | **Disagree** | **Neutral** | **Agree** | **Strongly Agree** |
| **Domain 1: Cultural Awareness** |  |  |  |  |  |
| 1. **I am aware of my own cultural values and biases.** |  |  |  |  |  |
| 2. **I recognize how my own cultural background influences the care I provide.** |  |  |  |  |  |
| 3. **I acknowledge cultural differences without being judgmental.** |  |  |  |  |  |
| 4. **I am willing to reflect on my assumptions about other cultures.** |  |  |  |  |  |
| 5. **I understand the importance of cultural awareness in patient safety and care.** |  |  |  |  |  |
| **Domain 2: Cultural Knowledge** |  |  |  |  |  |
| 1. **I have knowledge of health beliefs and practices of different cultural groups.** |  |  |  |  |  |
| 2. **I understand the influences of cultural traditions on health seeking behaviour.** |  |  |  |  |  |
| 3. **I am familiar with dietary practices of culturally diverse patients.** |  |  |  |  |  |
| 4. **I know how culture can influence communication styles and decision-making.** |  |  |  |  |  |
| 5. **I actively update my knowledge of cultural health needs through ongoing learning and training** |  |  |  |  |  |
| **Domain 3: Cultural Skills** |  |  |  |  |  |
| 1. **I assess patients’ cultural needs during admission and care planning.** |  |  |  |  |  |
| 2. **I use culturally appropriate communication methods with patients and families.** |  |  |  |  |  |
| 3. **I integrate patients’ cultural preferences into nursing care.** |  |  |  |  |  |
| 4. **I adapt health education materials to match cultural and language needs.** |  |  |  |  |  |
| 5. **I advocate for culturally competent care in my workplace.** |  |  |  |  |  |
| **Domain 4: Cultural Encounters** |  |  |  |  |  |
| 1. **I seek opportunities to interact with patients from diverse backgrounds.** |  |  |  |  |  |
| 2. **I learn from patients and families about their cultural values.** |  |  |  |  |  |
| 3. **I handle cultural misunderstandings respectfully.** |  |  |  |  |  |
| 4. **I use every encounter with diverse patients as a chance for learning.** |  |  |  |  |  |
| 5. **I am comfortable caring for patients from unfamiliar cultural groups.** |  |  |  |  |  |
| **Domain 5: Cultural Desire** |  |  |  |  |  |
| 1. **I am motivated to provide culturally competent nursing care.** |  |  |  |  |  |
| 2. **I am willing to adapt my care to respect patients’ cultural needs.** |  |  |  |  |  |
| 3. **I make an effort to reduce cultural barriers in healthcare.** |  |  |  |  |  |
| 4. **I am committed to continuous improvement in cultural competence.** |  |  |  |  |  |
| 5. **I believe culturally competent care improves patient outcomes.** |  |  |  |  |  |

**Scoring & Interpretation**

**Step 1: Add responses for all 25 items.**

**Step 2: Calculate mean score for each domain (total ÷ 5).**

**Step 3: Interpret results using the following guide:**

**Interpretation**

| **Mean Score** | **Interpretation of cultural Competence** |
| --- | --- |
| **1.0 – 2.9** | **Low Competence:-**Indicates major gaps in cultural knowledge and skills; immediate improvement and training needed |
| **3.0 – 3.9** | **Moderate Competence:-**Some cultural awareness and skills evident; requires targeted strengthening |
| **4.0 – 5.0** | **High Competence:-** Demonstrates strong or excellent cultural competence in nursing care |
