# Supplementary material for: Development and Validation of a Culturally Competent Care Module and Its Effect on Nurses’ Cultural Competence and Patient Satisfaction: Protocol for a Randomized Controlled Trial in a Tertiary Care Hospital in India
Source: JMIR Res Protoc. 2026 Jul 29;15:e84015. doi: 10.2196/84015 (PMC13469827; doi:10.2196/84015)
Supplement: Supplementary file 2 [file resprot_v15i1e84015_app2.docx]

**Introduction:**

1. **Greeting and Thank You**

"Good [morning/afternoon]. Thank you for taking the time to participate in this interview. My name is **Ligy** and I’m conducting research to develop a culturally competent care module for nurses, aimed at improving patient care satisfaction across diverse cultural contexts. Your insights will be invaluable in creating an effective and meaningful module."

2. **Purpose of the Interview**

"The goal of this interview is to gather perspectives from key stakeholders like yourself on what cultural competence means in nursing practice and what elements should be included in the module. This interview will take about [10 to 15mts) minutes, and your responses will be kept confidential."

3. **Permission to Record**

"To ensure I capture your responses accurately; I’d like to record this interview. The recording will only be used for research purposes, and no personal identifiers will be disclosed. Are you comfortable with this?"

**Interview Questions**

1. **Understanding Cultural Competence**
   - "In your view, what does cultural competence in nursing mean? Specifically, what qualities or skills should a culturally competent nurse possess to deliver effective care to patients of diverse backgrounds?"
2. **Specific Needs for Cultural Competence Training**
   - "From your perspective, are there specific cultural considerations, practices, or challenges unique to Hindu, Muslim, or Christian patients that nurses should be aware of? How can these be addressed in training to help nurses provide respectful and sensitive care?"
3. **Essential Module Content**
   - "What topics, skills, or practices do you feel are essential to include in a cultural competence training module for nurses? Can you suggest any practical exercises or learning activities that would be beneficial?"
   - How can this CCM be embedded into our induction program or translated from Bench to Bedside???

**Probing Questions (if needed)**

- "Could you elaborate on that point?"
- "Why do you feel this aspect is particularly important?"
- "Can you share an example that illustrates your point?"

**Closing Remarks**

"Thank you once again for sharing your valuable insights. Your contributions will play a crucial role in helping us create a module that meets the needs of both nurses and patients from diverse cultural backgrounds. If you have any further thoughts or would like to add anything later, please feel free to reach out."
